# Preventing and treating medication overuse headache

**DOI:** 10.1097/PR9.0000000000000612

**Published:** 2017-07-26

**Authors:** Karl B. Alstadhaug, Hilde K. Ofte, Espen S. Kristoffersen

**Affiliations:** a Department of Neurology, Nordland Hospital Trust, Bodø, Norway; b Institute of Clinical Medicine, The Arctic University of Tromsø, Tromsø, Norway; c Department of General Practice, Institute of Health and Society University of Oslo, Oslo, Norway; d Department of Neurology, Akershus University Hospital, Lørenskog, Norway

**Keywords:** headache, chronification, medication overuse headache (MOH)

## Abstract

Medication overuse headache is a secondary headache—a worsening of a pre-existing headache (usually a primary headache) owing to overuse of one or more attack-aborting or pain-relieving medications.

**Key Points**

According to the current concept, medication overuse headache (MOH) is a secondary headache—a worsening of a pre-existing headache (usually a primary headache) owing to overuse of one or more attack-aborting or pain-relieving medications.
Medication overuse headache has a prevalence of around 1% to 2% in the general population and should be suspected in anyone presenting with chronic headache (headache >14 days per month).
Migraine is the underlying headache disorder in most of the cases.
Existing criteria (International Classification of Headache Disorders–3 beta) often make straightforward diagnosis, but controversies around these criteria exist, and other chronic headache disorders may sometimes be difficult to rule out.
Any immediate relief medication has the potential to cause MOH.
Treatment guidelines for MOH are based on expert consensus and include withdrawal strategies, treatment of withdrawal headache, and eventually prophylactic medication for the underlying headache.

The concept that headache treatment per se may be the cause of chronification of headache is not new,^3^ but our understanding of this concept, and the terminology defining it, have changed throughout the years. The criteria for MOH are based on expert consensus rather than formal evidence. According to the current definition in the latest (2013) International Classification of Headache Disorders–3 beta, MOH is essentially defined as headache on 15 days or more per month that is regarded as a consequence of regular overuse of headache medication in a patient with a pre-existing headache.^18^ The classification does not include the number of drug units or dosage used, but the number of days per month the drug is taken.

Whether medication overuse is a separate secondary headache rather than a complication of a primary chronic headache (migraine or tension-type headache)^14^ is still debated. Medication overuse headache can occur in patients with an underlying secondary headache, but only exceptionally.^11^ All abortive headache medications taken regularly may lead to MOH.^9,30^ However, MOH must not be confused with immediate or delayed headache secondary to use of, exposure to, or withdrawal from other substances such as nitroglycerin, histamine, or caffeine.^18^

Reducing the dose and eventually discontinuing the overused medication are the natural first choices of treatment, but experts differ on the best way to do so. Among patients who manage to discontinue the overused medication, 50% to 70% will revert to an episodic headache pattern.^9,11^

In this Pain Clinical Update, we present MOH as a prevalent condition with huge individual and societal consequences, focus on the clinical aspects of this condition, and emphasize that it can be prevented and treated. This article is based on an unsystematic search in PubMed for criteria, guidelines, trials, and reviews of MOH followed by a discretionary selection of publications. Some of the references used as “clinical evidence sources” are commented on in the reference list.

## 1. Prevalence and impact on individuals and society

Medication overuse headache is a global health problem with a prevalence in the general adult population of different countries ranging from 0.5% to 7.6%.^10,11,32^ Robust data from Scandinavia indicate a prevalence of 1% to 2%, representing around 50% of all patients with chronic daily headache ([CDH], headache occurring on 15 or more days per month for more than 3 months). There is a clear preponderance of women.^17,19,31^ Compared with no headache or episodic headache, CDH is more detrimental to one's health, causing significant reductions in quality of life and workplace productivity. Comorbid psychiatric disorders and other associated stressors may have a negative effect on these measures, but data also indicate that medication overuse adds considerably to patients' problems.^22^

## 2. Risk factors

Most of what we know about the risk factors for MOH derives from cross-sectional studies, which makes it difficult to ascertain whether an identified factor is a cause or an effect of chronic headache. Medication overuse headache is more common in women, with a male to female ratio of 1:3–4.^20^ Prevalence is highest in the fourth decade of life, seemingly decreasing thereafter. Associations have been found between MOH and low income and educational levels, perhaps indicating either a cause of the disease or a result of living with a disabling condition. In addition, the literature notes associations between MOH and smoking, sleep disturbances, and high body mass index. Depression and anxiety are more prevalent in patients with MOH than in those with episodic migraine. The risk of developing MOH seems to be greater in persons with a family history of MOH or substance overuse,^4^ suggesting some degree of inheritance (social or biological).

A pre-existing primary headache, which is obligatory for a diagnosis of MOH, can also be regarded as an important risk factor. Migraine is an underlying disorder in around 60% to 80% of patients,^9,12,19,23^ and some authors suggest that pathophysiological mechanisms involved in migraine are also present in MOH.^9^ As expected, frequent headache at baseline is associated with a higher risk of developing MOH than infrequent headache, and persons with a daily or almost daily intake of analgesics (for any condition) are more likely to develop chronic headache 11 years later.^17^ A population-based study in the United States showed that most patients with CDH reported using caffeine-containing over-the-counter combination analgesics, triptans, or opioids, whereas they reported using aspirin and ibuprofen less frequently,^26^ suggesting that aspirin and ibuprofen are less likely to cause CDH. A study from 2002 indicated that triptans may cause MOH more rapidly and at lower dosages than simple analgesics and ergots.^23^ These results came from retrospective data in a study conducted at a specialized headache center, which casts doubt on the generalizability of the results. A recent systematic review found that the rate of MOH associated with analgesics and opioids in patients with episodic migraine was considerably higher than the rate of MOH associated with triptans and ergots.^30^ These seemingly contradicting results may reflect differences in drug availability and treatment cultures in different parts of the world.

In a population-based study from Norway, which included more than 25,000 participants, regular use of tranquilizers at baseline, or the combination of chronic musculoskeletal complaints, gastrointestinal complaints, and a Hospital Anxiety and Depression Scale score >11, was associated with a more than 5-fold in-creased risk of MOH 11 years later.^17^ These variables were also risk factors for developing CDH without medication overuse. Interestingly, smoking and physical inactivity at baseline more than doubled the risk of MOH 11 years later but did not increase the risk of CDH without medication overuse.

## 3. Pathophysiology

Given that medication is the cause of the chronic headache in MOH, and that a pre-existing headache is required, there is no reliable animal model for this disorder. In view of other risk factors for developing MOH, such as psychosocial and socioeconomic factors, the pathophysiological mechanisms must be very intricate. Given that migraine is the strongest associated disorder, the mechanisms involved in migraine and its chronification form much of the basis for our understanding of MOH. Imaging studies (reflecting changes in central pain networks), neurophysiological studies (indicating neuronal hyperexcitability), and evidence of sensitization of both peripheral and central nociceptive pathways in animals exposed to pain medication have shed light on the complex pathophysiology of MOH,^9^ but a clear picture has not emerged. Polymorphism in several genes, such as those encoding angiotensin-converting enzyme, brain-derived neurotrophic factor, catechol-O-methyltransferase, and serotonin transporter, has been linked to MOH, but such associations should be approached with caution. Common complex disorders, such as MOH, are unlikely to be caused by a locus of large effect, and the magnitude of the effect that a candidate gene exerts on the disease is very often overestimated. When researchers applied the DSM-IV to MOH patients, they found that two-thirds fulfilled the criteria for dependence.^24^ In line with the concept that patients with MOH share similar characteristics to persons with addiction,^7^ some authors have suggested that mechanisms involved in dependence processes also apply to MOH.^13^

## 4. Diagnostics

**Figure 1.**
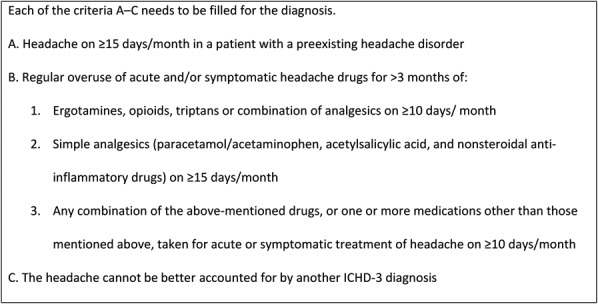
Diagnostic criteria for medication overuse headache, adapted from International Classification of Headache Disorders–3 beta^18^ (diagnoses 8.2.1 to 8.2.8). Adaptations are themselves works protected by copyright. So, to publish this adaptation, authorization must be obtained both from the owner of the copyright in the original work and from the owner of copyright in the translation or adaptation.

Clinicians should always suspect MOH in a patient who reports frequent headaches, especially if there is a previous history of migraine. It is important to note that the headache in MOH has no unique features.^23^ Many patients use relief medication on a more or less regular basis. A switch to a preemptive use of drugs, treating the anticipation of the headache rather than the headache itself, should cause concern. Lack of typical headache characteristics may cause diagnostic challenges, but if we apply the criteria in Figure 1, diagnosis is usually straightforward. However, other chronic headache conditions such as chronic migraine and chronic tension-type headache may be the cause; again, the “chicken or the egg” dilemma arises. Is the overuse of drugs a cause or a consequence? This problem may be approached pragmatically. The association between “overuse” and worsening of a pre-existing headache is based on an assumption, and improvement after discontinuation of medication overuse is no longer required to make a diagnosis of MOH. Our understanding of MOH is based on the idea that there must be an underlying (primary) headache to develop it, and that there is also a secondary cause, and therefore the patient receives both a primary and a secondary headache diagnosis, such as MOH and chronic migraine. If drug withdrawal converts a chronic condition to an episodic one, the MOH diagnosis was probably accurate. If it remains chronic, the MOH diagnosis could have been wrong, but not necessarily so. Conceivably, in some patients, medication overuse may have lasting harmful effects on headache, and hence may be a causal factor even when discontinuation does not reduce headache frequency. In any case, the condition is a cause for concern because long-term overuse of even simple analgesics may result in serious side effects. Other secondary headaches, such as those caused by increased intracranial pressure, should not be overlooked. Thus, on follow-up, it may be important to review the headache diagnosis. A particular problem that may arise is known as “new daily persistent headache,” which is a headache that is unique in the sense that it is daily from onset, unremitting within 24 hours, and long-lasting. It occurs typically, but not exclusively, in individuals without a previous history of headache.

Clinical neurological examination of a patient with MOH is recommended and should be normal. Keeping a headache diary with detailed information about medication use for a minimum of 4 weeks is a very helpful tool to document MOH. Clinicians should order brain imaging only if they suspect another secondary headache. When deciding how to treat the patient, we may find it useful to categorize patients into 2 groups^25^: relatively uncomplicated cases (type I, no behavioral impairment and no overuse of barbiturates or opioids) and complicated cases (type II, significant psychological issues and/or overuse of barbiturates and/or opioids). We may also regard patients with previous withdrawal failure as complicated cases.

## 5. Treatment and prevention

**Figure 2.**
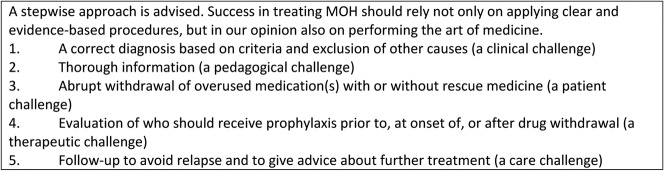
General current treatment recommendations.

There is no universal consensus on how to treat MOH, and we lack evidence from sufficiently powered randomized clinical trials (RCTs) to recommend one specific treatment approach^8^ (Fig. 2). Tertiary care centers often report high rates of treatment success, but the generalizability of these results is questionable owing to selection of patients with high motivation, exclusion of patients with prior detoxification failures and comorbid psychiatric illnesses, high dropout rates, and uncontrolled trials without intention-to-treat analyses. Patients with MOH are heterogenous, from individuals with migraine without comorbidity who are overusing a single medication to individuals with a mixture of headaches, massive comorbidity, behavioral dependence, and overuse of several drugs. Furthermore, studies indicate that such differences are partly culturally conditioned, and they also show that drugs are overused when they are easily accessible and inexpensive.^12^

## 6. Drug withdrawal

Most experts now regard withdrawal of the overused medication as the treatment of choice, as it often leads to improvement of the headache.^5^ In general, around 50% to 70% of patients with MOH seem to respond well to withdrawal therapy, and no withdrawal regime seems to be superior.^8,16^ European guidelines from 2011, based on publications with a low level of evidence and on expert consensus, state that early discontinuation of overused medications is the first line of management.^11^ Tapered withdrawal is recommended for benzodiazepines, opioids, and barbiturates, but in other cases abrupt withdrawal is recommended, with^2^ or without^11^ another medication to ameliorate withdrawal symptoms.

## 7. Withdrawal symptoms

Many patients experience withdrawal symptoms lasting 1 to 2 weeks. The most common symptom is an initial worsening of the headache, accompanied by various degrees of nausea, vomiting, hypotension, tachycardia, sleep disturbance, restlessness, anxiety, and nervousness.^11^ As there is no established optimal withdrawal method for MOH, many different strategies have been suggested, including antiemetics and neuroleptics to reduce abstinence-like symptoms, intravenous administration of ergotamines, and substitution of an another type of analgesic (rescue medication). Steroid treatment does not seem to be more effective than placebo in the acute withdrawal phase.^8^

## 8. The withdrawal process

For many uncomplicated patients, advice to discontinue the overused medication may be sufficient, with success rates of up to 70%.^9^ The results from a multicenter study (2 centers in Latin America and 4 in Europe) testing the effect of a multinational consensus protocol for management of uncomplicated MOH were reported recently.^29^ Approximately two-thirds of the 376 study patients were no longer overusing medication, and almost 50% had reverted to an episodic headache pattern, at the end of the study. Our clinical experience shows that most patients overusing simple analgesics and triptans, as well as combination analgesics containing codeine, manage abrupt withdrawal without tapering. However, it is extremely important to inform patients about the withdrawal symptoms and tell them that headache often becomes worse before getting better. Adding preventive medication at the outset is at least as effective as, and causes less headache suffering than, withdrawal without such medication.^15^ Generally, avoiding additional medication is a good idea, and it seems that patients with uncomplicated (type I) MOH manage well without initial prophylactic medication. However, we recommend that physicians make individual assessments and come to a decision in collaboration with the patient. A systematic review of articles published between 2004 and 2014, which classified evidence in accordance with the American Academy of Neurology's *Clinical Practice Guideline Manual*, indicated a better outcome if onabotulinumtoxin type A and topiramate were used in patients with chronic migraine plus MOH.^5^ Post hoc analyses in studies of patients with chronic migraine have shown that topiramate and onabotulinumtoxin type A (8 d/mo vs 6 d/mo in the placebo group) can reduce headache frequency without medication withdrawal,^27,28^ but such practice is not in line with current guidelines, and these results do not seem superior to those of other studies with initial withdrawal. Given these results, the authors suggested discontinuation of the overused medication with the addition of preventive medication, but they called for more adequately sized RCTs. According to the European^11^ and Danish guidelines,^1^ individualized preventive medication can be started before or on the first day of withdrawal, but it can also be postponed until the result of withdrawal is clear. In general, preventive therapy in MOH with amitriptyline, flunarizine, beta-blockers, valproic acid, cannabinoids, pregabalin, acupuncture, and occipital nerve stimulation either failed or had too little scientific evidence to be recommended.^9^

There seems to be no difference in the efficacy of inpatient or outpatient withdrawal management.^6^ Therefore, outpatient management should probably be offered to most patients, but it is considered a good practice point to offer inpatient treatment to patients with type II MOH.^11^ On a global basis, most people with headaches are primarily self-treating or are followed up by general practitioners (GPs). Medication overuse headache requires no high-technology investigations and may therefore be diagnosed and managed by any skilled physician. Since most MOH patients have been in contact with a GP, and almost half have had such contact in the previous year, primary care is probably a suitable area for the prevention and treatment of MOH. A brief intervention, consisting mainly of advice to MOH patients, was found highly effective in a recent RCT from a Norwegian primary care setting.^21^ Treating type I MOH in primary care may free more resources for referrals to headache specialists for the most complicated patients. This consideration is important, especially in countries with less access to secondary care specialists.

## 9. Prevention

Given the high prevalence of migraine and tension-type headache, and the fact that virtually anyone with such a disorder may be at risk of developing MOH, the number of people at risk is substantial. Educational strategies to increase knowledge among health care providers to identify patients at risk and inform their patients about the risks and modify their headache management habits, information campaigns to raise public awareness, and warning labels on over-the-counter analgesics are possible preventive strategies. As specific modifiable risk factors for MOH may exist, other measures such as restrictions on the use of tranquilizers and tobacco and increased physical activity may also be effective.^17^

## 10. Prognosis

Studies have reported relapse rates of 20% to 60% (with the majority between 25% and 35%) of patients within the first year after withdrawal, but few relapses after 12 months.^9,20^ However, as most follow-up studies are conducted in tertiary care centers, the generalizability of these results may be questioned. Furthermore, the results of these relapse studies should be compared with caution because they varied both in the use of headache classifications and criteria for improvement.

Studies support that initially detoxified patients can be followed up by their GP.^20^ The literature is conflicting on whether there are prognostic factors that may predict successful treatment without relapse in the long term.

## 11. Conclusions and recommendations

Medication overuse headache can be both prevented and treated, but it is not always an easy task. Outpatient withdrawal should probably be offered to most patients, but complicated cases may require inpatient treatment. A recent trial with a consensus protocol^29^—including (1) advice on medication withdrawal, (2) early discontinuation with supportive treatment for withdrawal headache, (3) optional early preventive medication, (4) symptomatic treatment using a different medication than the one previously overused, and (5) follow-up over a 6-month period—supports existing recommendations^1,2,5,9,11^ that most patients will benefit from a stepwise combination of advice, withdrawal of overused medications, prophylaxis, and regular follow-up (Fig. 2).

## Disclosures

The authors have no conflict of interest to declare.
